# Neural Decision Boundaries for Maximal Information Transmission

**DOI:** 10.1371/journal.pone.0000646

**Published:** 2007-07-25

**Authors:** Tatyana Sharpee, William Bialek

**Affiliations:** 1 Crick-Jacobs Center for Theoretical Biology and Laboratory of Computational Neurobiology, The Salk Institute for Biological Studies, La Jolla, California, United States of America; 2 Joseph Henry Laboratories of Physics, Lewis–Sigler Institute for Integrative Genomics, The Princeton Center for Theoretical Physics, Princeton University, Princeton, New Jersey, United States of America; Lund University, Sweden

## Abstract

We consider here how to separate multidimensional signals into two categories, such that the binary decision transmits the maximum possible information about those signals. Our motivation comes from the nervous system, where neurons process multidimensional signals into a binary sequence of responses (spikes). In a small noise limit, we derive a general equation for the decision boundary that locally relates its curvature to the probability distribution of inputs. We show that for Gaussian inputs the optimal boundaries are planar, but for non–Gaussian inputs the curvature is nonzero. As an example, we consider exponentially distributed inputs, which are known to approximate a variety of signals from natural environment.

## Introduction

What we know about the world around us is represented in the nervous system by sequences of discrete electrical pulses termed action potentials or “spikes” [1]. One attractive theoretical idea, going back to the 1950s, is that these representations constructed by the brain are efficient in the sense of information theory [2]–[4]. These ideas have been formalized to predict the spatial and temporal filtering properties of neurons [5]–[9], as well as the shapes of nonlinear input/output relations [10], showing how these measured behaviors of cells can be understood as optimally matched to the statistical properties of natural sensory inputs. There have been attempts, particularly in the auditory system, to test directly the prediction that the coding of naturalistic inputs is more efficient [11]–[15], and this concept of matching has been used also to predict new forms of adaptation to the input statistics [16]–[21]. Despite this progress, relatively little attention has been given to the problem of optimal coding in the presence of the strong, threshold–like nonlinearities associated with the generation of spikes [22].

Sensory inputs to the brain are intrinsically high dimensional objects. For example, visual neurons encode various patterns of light intensities that, upon moderate discretization, become vectors in 10^2^–10^3^ dimensional space [23], [24]. We can think of the “decision” to generate an action potential as drawing boundaries in these high dimensional spaces, so that a theory of optimal coding for spiking neurons is really a theory for the shape of these boundaries. In the simplest perceptron-like models [25], boundaries are planar, and spiking thus is determined by only a single (Euclidean) projection of the stimulus onto a vector normal to the dividing plane. In the perceptron limit, the optimal choice of decision boundaries reduces to the choice of an optimal linear filter. But a number of recent experiments suggest that neurons, even in early stages of sensory processing, are sensitive to multiple stimulus projections, with intrinsically curved decision boundaries [17], [24], [26]–[30]. Here we try to develop a theory of optimal coding for spiking neurons in which these curved boundaries emerge naturally.

We consider a much simplified version of the full problem. We look at a single neuron, and focus on a small window of time in which that cell either does or does not generate an action potential. We ignore, in this first attempt, coding strategies that involve patterns of spikes across multiple neurons or across time in single neurons, and ask simply how much information the binary spike/no spike decision conveys about the input signal. Let this input signal be a vector **r** in a space of *d* dimensions and let the distribution of these signals be given by *P*(**r**). Note that what we call “the input signal” could in fact reflect the recent history of the physical inputs; we are interested in all aspects of the input that are (potentially) relevant to the question of whether a single neuron will generate an action potential in some small window of time. If the binary output of the neuron is *μ*, we are interested in calculating the mutual information *I*(*μ*,**r**) between *μ* and the input **r**.

## Analysis

We can always write the information as a difference between two entropies [1], [31], the response entropy and the noise entropy: *I*(*μ*,**r**) = *H*_response_−*H*_noise_. This expression holds for any model of neural noise and response probability [31]. Differences between models for neural noise and response generation will affect particular expressions for these two terms. In our simplified problem, with a single neuron giving binary responses, the response entropy,
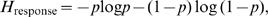
(1)is completely determined by the average spike probability *p*. We might imagine that this probability is set by constraints outside the problem of coding itself. For example, generating spikes costs energy, and so metabolic constraints might fix the mean spike rate [32]–[35]. Our problem, then, is to find coding strategies that minimize the noise entropy at fixed *p*.

**Figure 1:**
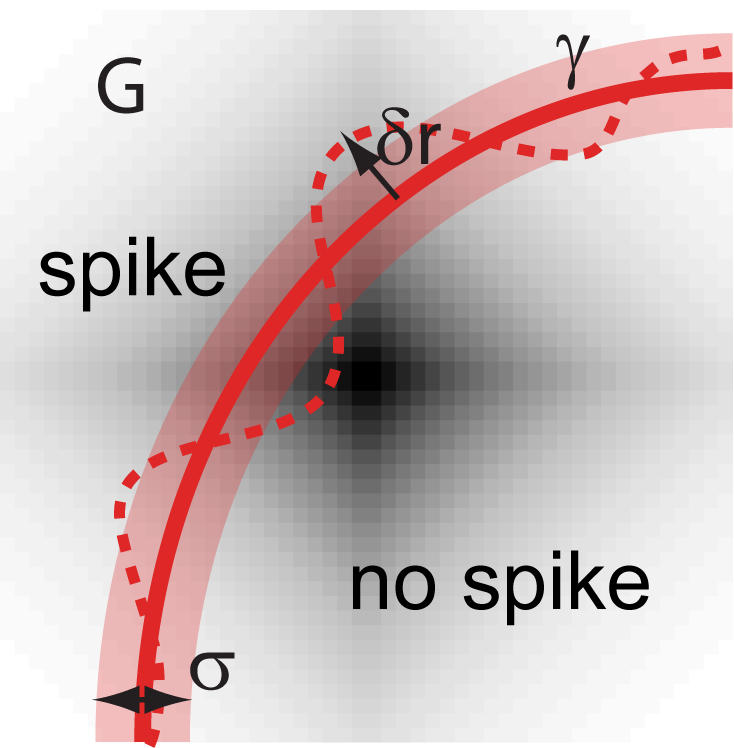
Schematic illustration of a hypothetical decision boundary relative to the input probability distribution, shown as a color plot. In this case, the decision boundary (red solid line) is shown as extending to infinity, but closed contours are also possible. Variations in contours' shape (as illustrated with a dashed curve) not only shift the position of the decision boundary relatively to the input probability distribution, but also change the overall length of the contour and its infinitesimal arc length element.

In the absence of noise, the coding scheme which maps signals into spikes (or their absence) is a boundary in the *d*–dimensional space of inputs. Figure 1 illustrates a hypothetical coding scheme for two-dimensional inputs. In general, stimuli do not explore the input space uniformly. In Figure 1 we illustrate one example probability distribution as a color-plot, with darker values for more common inputs and whiter values for more rare inputs. The probability distribution is normalized to sum to 1 over all of the possible inputs. Therefore, if spikes mostly occur in some domain *G* (the boundary of this region is shown with a solid red line in Figure 1), then the average spike probability *p* would just equal to the probability that inputs would fall within the spike region: 
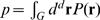
. In the absence of neural noise, all codes with the same value of *p* would transmit the same amount of information, and there would be an infinite set of nominally optimal domains *G*.

 We will work in an approximation where the noise is small. We will allow for the noise magnitude to vary with the stimulus to account for the fact that, for example, noise level could be larger for inputs of large magnitude. Then if the boundary of the spiking domain is some (*d-1* dimensional) surface *γ*, as illustrated by a solid red line in Figure 1, we expect that responses far from this boundary are essentially deterministic and do not contribute to the noise entropy; all of the contributions to *H*_noise_ should arise from a narrow strip surrounding the boundary *γ*. Within this strip, the response is almost completely uncertain. Thus we can approximate the noise entropy by saying that it is ∼1 bit inside the strip, and zero outside; the total noise entropy is then the mass of probability inside the strip. The width of the strip is proportional to the strength of the noise, and if noise is small the probability distribution of inputs does not vary significantly across this width. Thus, we can write the overall noise entropy as an integral along the decision boundary *γ*:
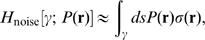
(2)where *ds* is the infinitesimal surface element of dimension *d-1* on the decision boundary *γ* and σ(**r**) is the amplitude of the noise for inputs at location **r**. The exact shape of the nonlinear function describing how spike probability changes across the domain boundary might introduce additional numerical factor of order unity in Eq. (2), but these factors can always be accounted for by defining σ to be the *effective* noise level.

While our choice of threshold–like transitions between spiking and non–spiking regions considerably narrows the types of possible input-output transformations, it still leads, as we show below, to highly nontrivial, yet tractable, solutions. We will treat the local noise length scale σ(**r**) as a pre-defined function; it can take arbitrary positive values and will set the units for locally measuring contours' curvature and 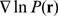.

Taking into account that the response entropy *H*_response_ only depends on the average spike probability, the optimal contour providing maximal information may be found by minimizing
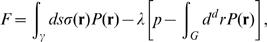
(3)where λ is the Lagrange multiplier incorporating the constraint for the average spike probability *p*. To find an optimal contour, we look for a contour such that the functions *F* would not change, to the first approximation, under small perturbations *δ* **r** in the contour shape, cf. Figure 1. Two effects take place with any perturbation of the contour. First, the contour will now be positioned at slightly different points, so that there will be a change in the values of input probability distribution that contribute to the functional *F*. This effect contributes a term 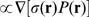 to the first order variation in the value of *F*. The second effect of perturbing a contour is that the overall length of the contour changes. This effect can be quantified locally through a change in the arc length element 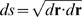. For two-dimensional inputs, cf. Figure 1, the arc length element changes with perturbation by a factor 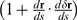. It can be verified that only perturbations along the contour's normal could possible change the value of the functional. Then, the change in the arc length element can be written as a dot product between the tangent vector to the contour 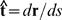 and the change in the direction of the normal 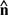 of the contour along the contour, 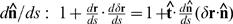. One might recognize here the expression for the curvature, 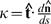 [36]. Thus, a first-order change in the arc length (and overall length of the contour) is observed only for curved contours. In the case of straight lines, for example, there is no first-order change in the arc length element. This result can be generalized to inputs of arbitrary dimensionality by taking into account that (i) now there will be a set of tangent vectors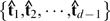 defining the tangent plane, and that (ii) the change in the surface element is affected by a change in the direction of the normal 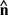 to the contour along all of tangent vectors, 
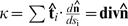
, where *κ* is the total curvature of the decision boundary *γ*. Putting it all together, we find that the first order variation in the functional F for multidimensional inputs is given by.
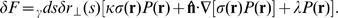
(4)Because perturbations at various points along the surface are independent, the optimal contour should satisfy:
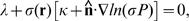
(5)


First, let us consider the simplest case where inputs are uniformly distributed and noise level is constant. In this case, the optimal contours obtained according to Eq. (5) are circles. The circle radius is determined by the average firing rate, which in this case equals its area. The fact that a circle turns out to be an optimal solution for uniform inputs is, perhaps, not surprising. After all, the optimization problem we consider is related to the theory of minimal surfaces, which have the smallest circumference for a given area. A circle is the most obvious example of a minimal surface. In the context of information transmission, fixing the average firing rate is equivalent to fixing the enclosed area, whereas minimizing noise entropy is equivalent to minimizing the circumference in the case of minimal surfaces. Despite its simplicity, the finding that optimal decision contours are circles for uniform probability distribution indicates possible functional advantages of the circular symmetry observed for receptive fields in the retina. After all, in the case of retinal processing, the probability to have certain intensity value is uniform across space. Below we solve Eq. (5) to find optimal decision boundaries for two example non-uniform probability distributions: a Gaussian and the exponential. The exponential distribution is important not only as an example of non–Gaussian inputs, but also because it captures some of the essential statistical properties found in real–world signals [37], [38]. In these examples, we assume that the noise level does not depend on stimulus coordinate. With constant noise level, parameter *λ* can be rescaled by a factor of *σ*, so there is only one parameter in the problem.

Consider the case of uncorrelated Gaussian inputs 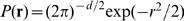, where the equation for optimal contours takes the form:
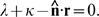
(6)The families of possible solutions, include circles [*λ = R-(d-1)/R*, where *R* is the circle radius] and straight lines *κ = *0 [*λ = R*, where *R*, in this case, is the smallest distance from the line to the origin]. Circles and straight lines turn out to be the only possible smooth contours. Other smooth contours are not possible because the mean curvature increases exponentially with distance 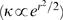, causing contours to self–intersect before a closed smooth contour can be obtained.

To choose between circles and straight lines, we calculate the noise entropy as a function of spike probability *p* in both cases. From Eq (2), we see that *H*_noise_ is proportional to the noise level σ, so in what follows we compute the noise entropy in these units. For a circle in two dimensions, the noise entropy is given by 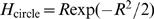. If we presume that spikes occur whenever inputs fall outside of the circle, the corresponding average spike probability is given by 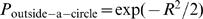. Thus, within the family of circular solutions, there is a one-to-one correspondence between the noise entropy and the average spike probability. It follows from Eq. (6) that the Lagrange multiplier is *λ = R−(d−1)/R*.

For straight lines a distance *R* from the origin, the noise entropy is given by 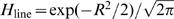 (the integral of the probability distributions with respect to component along the line gives 1). The spike probability associated with a line a distance *R* from the origin can be obtained by integrating the probability distribution from the line to infinity (on the side where spikes are thought to occur). This leads to an error function, with 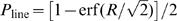. Thus, there is a one-to-one relationship between the threshold value *R* and the average firing rate *P*_line_. From Eq. (6), we find that the Lagrange multiplier λ = *R*. Knowing the threshold value *R,* one can then look up the corresponding value of the noise entropy *H*_line_. Therefore, similarly to the case of circular decision boundaries, within the family of planar threshold decisions there is also a one-to-one relationship between the average firing rate and the noise entropy.

**Figure 2:**
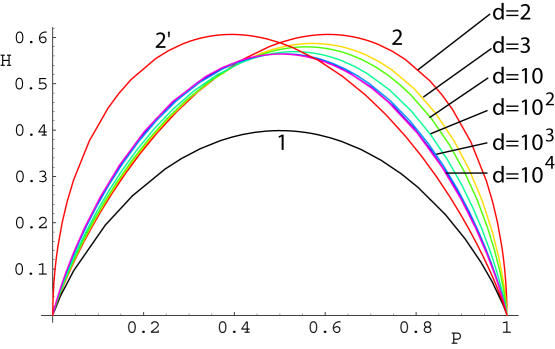
Comparison of noise entropies for straight line solutions (1) and circles with spiking on the outside (2) or inside (2

Comparing the noise entropy as a function of the corresponding average spike probability both the family of circular and linear solutions, we find that threshold decisions with respect to straight lines lead to smaller values of noise entropy, and therefore larger values of information transmitted, for all values of average spike probability, cf. Figure 2.

This result can be generalized to inputs of arbitrary dimensionality. Expressions for entropy and probability for straight lines do not change with dimensionality *d*, while the corresponding values for circles are:
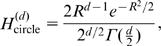
(7)

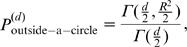
(8)where 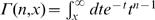 is the incomplete Gamma function. In Figure 2 we plot these solutions to show that for any probability *p* and dimensionality *d*, the optimal separation is with straight boundaries. This result also holds for correlated Gaussian inputs, where the optimal hyper-plane is the one which intersects the axis of largest variance and is parallel to other coordinate axes. Thus, the presence of correlations between input signals lifts the degeneracy of optimal solutions observed in the uncorrelated case. Whereas, in the case of white Gaussian inputs, any line with the same smallest distance *R* from the origin, provides equivalent encoding in terms of information transmission regardless of its orientation, the presence of correlations breaks the spherical symmetry, so that there is only one optimal decision line in the case of encoding correlated Gaussian inputs.

As an example of a non–Gaussian probability distribution, we consider an exponential distribution in two dimensions (2D): 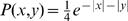. The local equation for optimal contours (5) can be written parametrically:
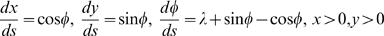
(9)where angle *φ* determines the tangent 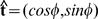 and normal 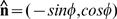 of the curve, as well as the curvature *κ = −dφ/ds*. Solutions in other quadrants can be obtained from Eq. (9) by an appropriate change of variables.

For *λ* = ±1, the family of optimal contours includes straight lines parallel to coordinate axes. Such straight lines represent 1D threshold decisions, and in this case the noise entropy equals the spike probability, decreasing exponentially with the threshold *R* for decision *x>R*:
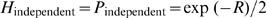
(10)The only other straight line solution that satisfies the optimality condition in Eq (9) is a line *y* = ±*x*; it corresponds to spike probability *p* = 1/2. Straight lines of the same angle that do not pass through the origin do not satisfy the optimality condition, but they provide a useful benchmark for other solutions in the middle range of probabilities 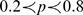, where they are better than the straight lines parallel to the axes: 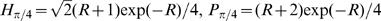, as illustrated in Figures below.

Within a single quadrant, the optimal solution can be found explicitly in terms of angle *φ* relative to the starting point where *φ = φ_0_*, *x_0_ = x*(*φ_0_)*, and y*_0_ = y*(*φ_0_)*: 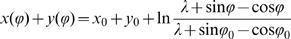


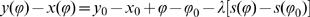
(11)where arc length *s*(*φ)* depends on the angle *φ* as:
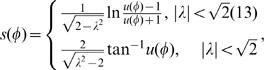
(12)

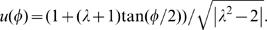
These solutions are similar to a logarithmic spiral for 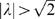, and to a hyperbola for 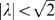, with asymptotes at 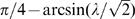 and 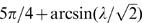. Asymptotes themselves are valid solutions within a quadrant; they will be part of a global solution. For all *λ*, the solution (12) within a quadrant intersects coordinate axes where it should be matched with similar solutions in other quadrants.

**Figure 3:**
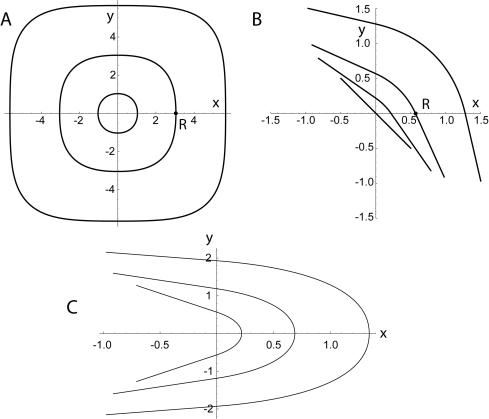
Optimal solutions for 2D exponential inputs: (A) closed “stretched circle” solutions are shown for *λ* = −0.3,−0.9,−0.99, numbers correspond to the increasing size of the curved segment throughout this legend. (B) Extended solutions symmetric around *y* = *x* line are shown for *λ*  = 0,−0.25,−0.5,−0.75. (C) Extended solutions symmetric around *x* = 0 line are shown for *λ = *0,−0.5,−0.75 [this type turned out to be suboptimal, albeit by a small margin, compared to either A or B, cf. Figure 4].

The possible types of global solutions are shown in Figure 3. They could be either closed (“stretched circles”; A) or extended (B and C). For the 2D exponential distribution, no curved solutions that extend to infinity and are confined to one or two quadrants can exceed the efficiency level of *H = P* achievable by straight lines parallel to the axes (11). This is due to the arc length factor 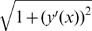 in the noise entropy 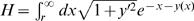, which is absent in 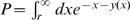, so that *H>P* for all such solutions. This argument does not apply to solution spanning three quadrants or four quadrants, shown Figure 3. For 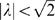, extended solutions can be formed by connecting asymptotes in two separate quadrants with a convex curve described by Eqs. (12,13). We will refer to such extended solutions as B or C depending upon whether the curved segment passes through one or two quadrants, cf. Figure 3. Extended solutions B are symmetric around *y = x* line, and exist only for −1<*λ*<0, while extended solutions C are symmetric around *x* = 0 line, and exist for −1<*λ*<1.

For all types of global solutions (A–C), boundary conditions specify a unique curve for each value of *λ*. In all cases, both entropy and probability can be found exactly as a function of *λ*. For solutions A we find
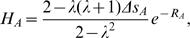
(13)

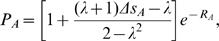
(14)where the arc length is given by 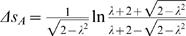, if 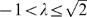, and 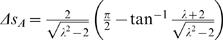, if 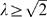. The size of the curved segment is 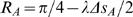. These solutions are continuous at 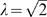.

**Figure 4:**
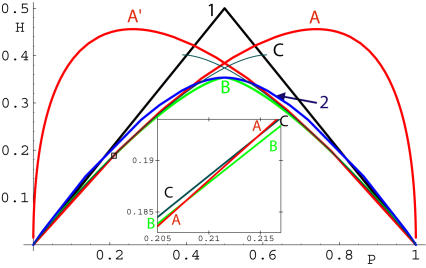
Noise entropy *H* along various decision boundaries with exponential inputs: “stretched circles” with spiking outside A or inside A′, extended solutions B and C; straight lines parallel to coordinate axes (1) and at ±π/4 angle (2). Solutions (A-C) and (1) satisfy the optimality Eq. (9), but not (2), which is optimal only at a single point at *p* = 1/2 where it becomes part of family of extended solutions B. Inset shows that switching occurs between solutions A and B.

For extended solutions B, the entropy and probability become
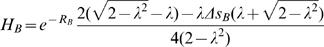
(15)

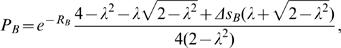
(16)where the arc length 
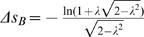
; and corresponding size of the curved segment is 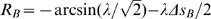. These solutions are valid only for −1< *λ* <0. More detailed calculations shows that solutions C are suboptimal compared to global solutions A or B; see Figure 4 and the discussion below. Note that neither A, B, nor C solutions exist for *λ*<−1.

The most physiologically relevant regime corresponds to *λ* = −1+*ε*, 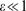. Here, all global solutions A-C have a large “radius” of the curved segment 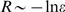. The probability and noise entropy depend exponentially on *R*, so that 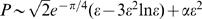 and 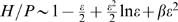. The constants *α* and *β* and depend on the solution type (A, B, or C). Because *β_A_< β_B_*< *β_C_*, solutions A are optimal for small *ε*. Near *p*≈0.2, intersections between the three curves occur. In the *O*(*ε^2^)* approximation, all of the three curves intersect at a single intersection point that splits into three once higher-order terms are included. As probability increases, B and C intersect first (A goes below), then A and B (the crossover point, C goes above), and finally, A and C (B goes below). The inset of Figure 4 shows A–B and A–C intersections. Thus, solutions A and B are optimal at extreme and medium probabilities, respectively. Solutions of type C are never optimal, and neither are the straight line solutions, except for the middle point *p* = 1/2.

### Conclusion

We have presented a general approach to finding optimal binary separations of multidimensional inputs. In the small noise limit, the curvature of the optimal bounding surface is determined locally by the probability distribution. While Gaussian inputs are optimally separated by hyper-planes, this is not the case in general. For example, in the case of exponentially distributed inputs in two dimensions, the optimal decision contours are curved and could either be closed or extended. Closed contours are optimal at extreme probabilities, while extended ones are optimal for spike probabilities near 1/2. The ubiquity of non–Gaussian signals in nature, particularly of the exponential distributions considered here, suggests that these results might be relevant for neurons across different sensory modalities.
